# Efficacy and safety of batroxobin in patients with acute ischemic stroke: A multicenter retrospective analysis

**DOI:** 10.1111/cns.14877

**Published:** 2024-08-04

**Authors:** Shu Liu, Shengyuan Wang, Qian Zou, Yinshuang Pu, Xiaobo Li, Zhenlun Hang, Zhiyou Cai, Chuanling Wang

**Affiliations:** ^1^ Department of Neurology Chongqing General Hospital Chongqing China; ^2^ Department of Neurology Southwest Jiaotong University Affiliated Hospital, Chengdu Third People's Hospital Chengdu China; ^3^ Chongqing Key Laboratory of Neurodegenerative Diseases Chongqing China; ^4^ Department of Neurology Yubei District Hospital of Traditional Chinese Medicine Chongqing Chongqing China; ^5^ Department of Neurology Banan District Hospital of Traditional Chinese Medicine Chongqing Chongqing China; ^6^ Chongqing Medical University Chongqing China

**Keywords:** acute ischemic stroke, batroxobin, functional outcomes, stroke recurrence

## Abstract

**Aims:**

The objective of this study was to evaluate the effectiveness of batroxobin in improving functional outcomes and reducing stroke recurrence among patients with acute ischemic stroke beyond the therapeutic time window for thrombolytic therapy.

**Methods:**

This multicenter, retrospective study enrolled 492 patients with acute moderate‐to‐severe ischemic stroke within 24 h. 238 patients were given standard (basic) therapy. On the basis of standard treatment, 254 patients received an initial intravenous infusion of batroxobin 10 U on day 1, followed by subsequent infusions of batroxobin 5 U on the 3rd and 5th days, respectively.

**Results:**

In the batroxobin group, 8.3% of patients experienced recurrence stroke, compared to 17.2% in the control group (HR, 0.433; 95% CI, 0.248 to 0.757; *p* = 0.003). Furthermore, intravenous batroxobin significantly improved the distribution of 90–120 day disability. Moderate‐to‐severe bleeding events were reported in three patients (1.2%) in the batroxobin group and one patient (0.4%) in the control group (*p* = 0.369).

**Conclusions:**

Among patients with acute moderate‐to‐severe ischemic stroke beyond the time window for thrombolytic therapy, treatment with intravenous batroxobin had a lower risk of stroke recurrence and a better recovery of function outcome without increasing bleeding events. Prospective studies are needed to further confirm.

## Graphical abstract

Intravenous batroxobin treatment was found to significantly reduce the risk of stroke recurrence and improve functional outcomes for patients with acute ischemic stroke beyond the thrombolytic therapy time window. Additionally, the treatment did not lead to an increase in moderate‐to‐severe bleeding events.

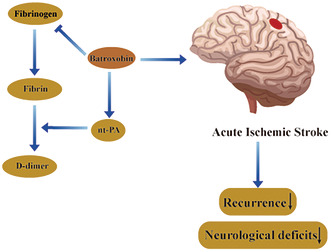

## 1 INTRODUCTION

Stroke is the second leading cause of death and the third most prevalent cause of disability worldwide.^1^ Reperfusion therapy including intravenous recombinant tissue plasminogen activator (rt‐PA) or endovascular treatment or intravenous rt‐PA bridging to endovascular treatment has been recommended as a standard treatment for ischemic stroke.^2^, ^3^ However, a significant portion of patients miss out on the optimal window for receiving reperfusion treatment due to delayed detection, and approximately half of patients do not benefit from timely initiation of acute reperfusion therapy.^4^, ^5^, ^6^ Therefore, potential treatment strategies are urgently needed in clinical practice. For secondary prevention after a stroke, the use of antiplatelet therapy for preventing secondary strokes has been widely recognized. Additionally, systematic reviews indicate that implementing secondary prevention measures, such as anticoagulants,^7^ statins,^8^ and antihypertensive therapies,^9^ effectively reduces the risk of secondary vascular events. However, despite these interventions, the risk of stroke recurrence for patients with acute ischemic stroke remains higher in the early stages of recovery (ranging from approximately 6% to 14% within 1 year).^10^

Batroxobin, a serine protease similar to thrombin that is derived from snake venoms, is widely utilized in the treatment of arterial thrombosis. Its binding affinity for fibrinogen is 10 times greater than that of thrombin.^11^ This interaction leads to a reduction in the amount of circulating fibrinogen, thereby inhibiting the formation of fibrin and elongation of thrombi. Prior in vivo studies of mice have demonstrated that batroxobin improves microcirculation, thereby protecting against severe ischemic tissue injury and accelerating vascular and skeletal muscular regeneration.^12^ Furthermore, several clinical studies showed that batroxobin reduced stroke recurrence and provided favorable outcomes, especially enhancing motor function in individuals suffering from acute ischemic stroke.^13^, ^14^

Based on these animal and small human studies, we carried out a multicenter retrospective study to assess the effectiveness and safety of batroxobin in treating acute moderate‐to‐severe ischemic stroke patients beyond the thrombolysis treatment time window.

## 2 METHOD

### 2.1 Study design and population

In this retrospective study, we included patients with acute ischemic stroke from September 2021 to October 2022 at three hospitals. Patients were eligible for the study if they were between the ages of 18 and 80, had an acute ischemic stroke with a National Institutes of Health Stroke Scale (NIHSS) score of 4–18 (range from 0 to 42, with higher scores indicating more severe stroke) within 24 h of symptom onset. The exclusion criteria were: (1) older than 80 years or younger than 18 years; (2) NIHSS score less than 4 or more than 18; (3) patients who had gastrointestinal bleeding within 6 months; (4) patients who have received intravenous thrombolytic therapy or mechanical thrombectomy; (5) patients who were on long‐term use of non‐steroidal anti‐inflammatory drugs; and (6) patients who had other serious diseases. This study was approved by the Chongqing General Hospital and conducted in accordance with the Declaration of Helsinki.

### 2.2 Treatments

Based on the treatment received, patients were divided into two groups: the batroxobin group (an initial intravenous infusion of 10 U of batroxobin plus 250 mL of normal saline on day 1, followed by subsequent infusions of 5 U of batroxobin on the 3rd and 5th days, respectively) or control group. Both groups also received antiplatelet therapy, lipid regulation, and management of risk factors that were followed according to guidelines.

### 2.3 Data collection

From patients' hospital electronic records, patient characteristics including demographics, laboratory examination, and comorbidities were collected at baseline. Patients were followed for 1 year after their treatment. The modified Rankin Scale (mRS) scores at 90–120 days of follow‐up were determined by trained site investigators/coordinators. An independent clinical‐event adjudication committee contacted patients or their families to ascertain if there had been a recurrent stroke and adverse reactions within 1 year.

### 2.4 Study outcomes

The primary efficacy outcome was stroke recurrence within 1 year. Additionally, the effects of treatment based on age, sex, history of smoking, history of hypertension, history of diabetes, history of coronary artery disease, history of stroke, history of fibrillation, history of peripheral arterial disease, history of high‐grade carotid stenosis (high carotid stenosis was defined as an intima thickness narrowing exceeding 70% through carotid ultrasound/magnetic resonance angiography, MRA/computed tomography angiography, CTA/digital subtraction angiography, DSA), NHISS score, and etiological stroke subtype were assessed in subgroup analyses.

Secondary efficacy outcomes were functional outcomes at 90–120 days measured using the modified Rankin Scale (mRS), with scores ranging from 0 (no symptoms) to 6 (death). This included the proportion of patients who did not have any disability (mRS score of 0 to 1), the proportion of patients who achieved functional independence (mRS score of 0 to 2), and the proportion of patients who were able to walk or look after themselves or better (mRS score of 0–3).

The safety outcome was defined as moderate‐to‐severe bleeding events, in accordance with the criteria outlined in the Global Utilization of Streptokinase and Tissue Plasminogen Activator for Occluded Coronary Arteries (GUSTO) guidelines.^15^ Fatal or intracranial hemorrhage or any hemorrhage that significantly affected a patient's hemodynamic status and required medical care was classified as severe bleeding. A moderate bleeding condition is characterized by bleeding that does not compromise hemodynamics, but still necessitates blood transfusion.

### 2.5 Statistical analysis

Patients in both groups presented similar baseline characteristics, with categorical variables presented using proportions and continuous variables presented using medians and interquartile ranges. Differences at baseline between groups were analyzed using the *χ* ^2^ test (or Fisher exact test when appropriate) on categorical data and nonparametric Wilcoxon rank‐sum test (or the independent sample Student's *t*‐test) on continuous variables. To assess the primary outcome, we employed the Kaplan–Meier method to estimate the cumulative risk of stroke recurrence at the 1‐year follow‐up. Additionally, we used a Cox proportional hazards model to estimate the hazard ratio (HR) and 95% confidence interval (CI). For secondary outcomes, we used a logistic regression model, adjusting for the following prespecified variables: age, sex, BMI, NIHSS, the proportions of anterior and posterior circulation infarctions, previous or current smoking, history of stroke, history of peripheral arterial disease, history of high‐grade carotid stenosis, and TOAST subgroups (etiological stroke subtype), to analyze the difference in functional outcome (mRS score) between the two groups. We reported the adjusted odds ratio (aOR) with 95% CI. A *p*‐value exceeding 0.05 was considered indicative of no significant difference between the two groups for all subsequent endpoints. Statistical analyses were conducted using IBM SPSS Statistics 26.0, while figures were generated using R software.

## 3 RESULTS

### 3.1 Patient population

**FIGURE 1:**
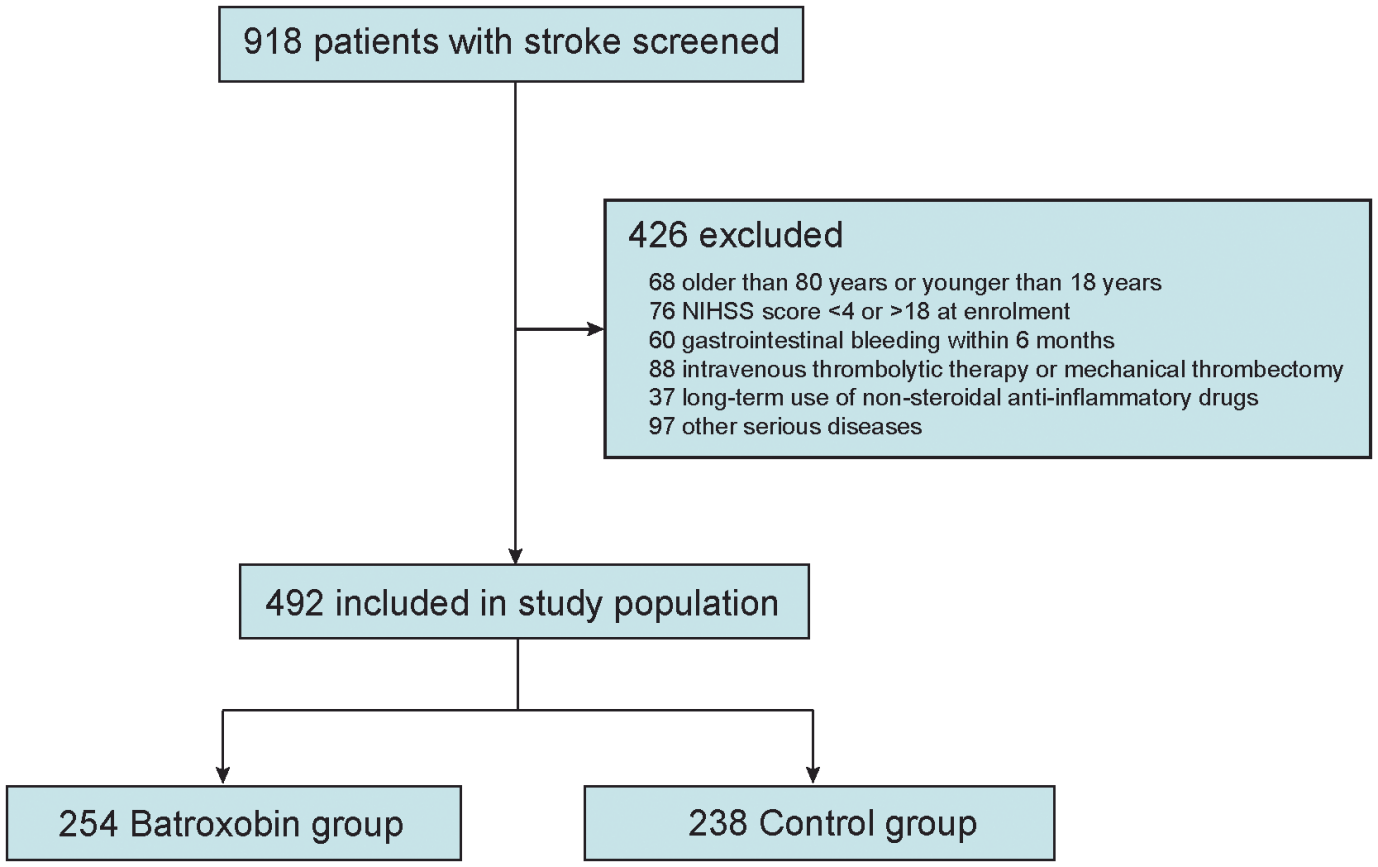
Study flowchart. NIHSS, National Institute of Health Stroke Scale.

Between September 1, 2021, and October 1, 2022, there were 918 patients included in this study, of which 492 patients (54.0%) met inclusion criteria. Of the eligible cohort, 238 (48.4%) were divided into batroxobin group, and 254 (51.6%) were divided into control group. A total of 426 patients did not meet the inclusion criteria. The reasons for exclusion included 68 patients who were older than 80 years or younger than 18 years, 70 patients who had an NIHSS score of less than 4 or more than 18, 60 patients who had gastrointestinal bleeding within 6 months, 88 patients who have received intravenous thrombolytic therapy or mechanical thrombectomy, 37 patients who were on long‐term use of non‐steroidal anti‐inflammatory drugs, and 97 patients who had other serious diseases (Figure 1). In both treatment groups, the baseline characteristics of the patients were similar (Table 1). Out of the 492 patients, the median age was 71 years with an interquartile range of 63–79, and 188 patients, accounting for 38.2%, were women. There were 36.8% of the patients with hypertension, 25.4% with diabetes, and 43.0% who were previous or current smokers.

**TABLE 1:** Baseline characteristics of the patients.

	Batroxobin group (*n* = 254)	Control group (*n* = 238)	*p*‐Value
Age, median (IQR), years	70 (64–79)	71 (63–79)	0.780
Sex			
Male	156 (61.4%)	148 (62.2%)	0.860
Female	98 (38.6%)	90 (37.8)	
SBP (mm Hg)	153 (139–169)	149 (135–164)	0.290
DBP(mm Hg)	90 (80–101)	88 (78–99)	0.520
BMI	22.6 (20.8–23.6)	22.8 (20.6–23.5)	0.930
TG CHOHDLLDL(mmol/L)			
TC	1.5 (1.05–2.26)	1.4 (1.0–2.0)	0.410
TG	4.93 (4.06–5.62)	4.69 (3.91–5.58)	0.080
HDL‐C	1.21 (0.97–1.48)	1.15 (0.96–1.42)	0.570
LDL‐C	2.90 (2.36–3.54)	2.53 (1.86–3.23)	0.090
Coagulation four indices			
PT	11.3 (10.5–12.6)	11.6 (10.8–12.9)	0.350
FIB	3.14 (2.57–3.83)	3.00 (2.51–3.59)	0.760
TT	17.6 (16.6–18.7)	16.9 (15.7–18.2)	0.120
APTT	26.4 (24.3–33.0)	29.5 (27.0–32.8)	0.230
Creatinine	69.0 (58.3–84.5)	71.0 (59.3–45.3)	0.070
Hematocrit	41.7 (39.0–45.5)	41.6 (38.‐15.3)	0.540
Previous or current smoking	64 (25.2%)	34 (14.3)	0.002
Medical history			
Hypertension	103 (40.6%)	78 (32.8%)	0.074
Diabetes	71 (28.0%)	54 (22.7%)	0.180
Coronary artery disease	112 (44.1%)	111 (46.6%)	0.571
Stroke	55 (21.7%)	33 (13.9%)	0.024
Atrial fibrillation	12 (4.7%)	20 (8.4%)	0.098
Chronic obstructive pulmonary disease	16 (12.9%)	9 (3.8%)	0.204
Peripheral arterial disease	47 (18.5%)	69 (29.0%)	0.006
High‐grade carotid stenosis	82 (32.3%)	111 (46.6%)	0.001
Time from onset to thrombolysis (≥6 h)	254 (100.0%)	238 (100.0%)	
NIHSS score			
<10	227 (89.4%)	212 (89.1%)	0.916
≥10	27 (10.6%)	26 (10.9%)	
The proportion of anterior/posterior circulation infarction			
Anterior circulation infarction	203 (79.9%)	196 (82.4%)	0.491
Posterior circulation infarction	51 (20.1%)	42 (17.6%)	
Etiological stroke subtype			
Large‐artery atherosclerosis	122 (48.0%)	94 (39.5%)	0.057
Small‐artery occlusion	23 (9.1%)	17 (7.1%)	0.438
Cardioembolism	83 (32.7%)	52 (21.8%)	0.007
Other	26 (10.2%)	75 (31.5%)	<0.001

Abbreviations: APTT, activated partial thromboplastin time; BMI, body mass index; DBP, diastolic blood pressure; FIB, fibrinogen; HDL‐C, high‐density lipoproteincholesterol; LDL‐C, low‐density lipoprotein cholesterol; NIHSS, National Institute of Health stroke scale; PT, prothrombin time; SBP, systolic blood pressure; TC, total cholesterol; TG, triglyceride; TT, thrombin time.

### 3.2 Outcomes

**FIGURE 2:**
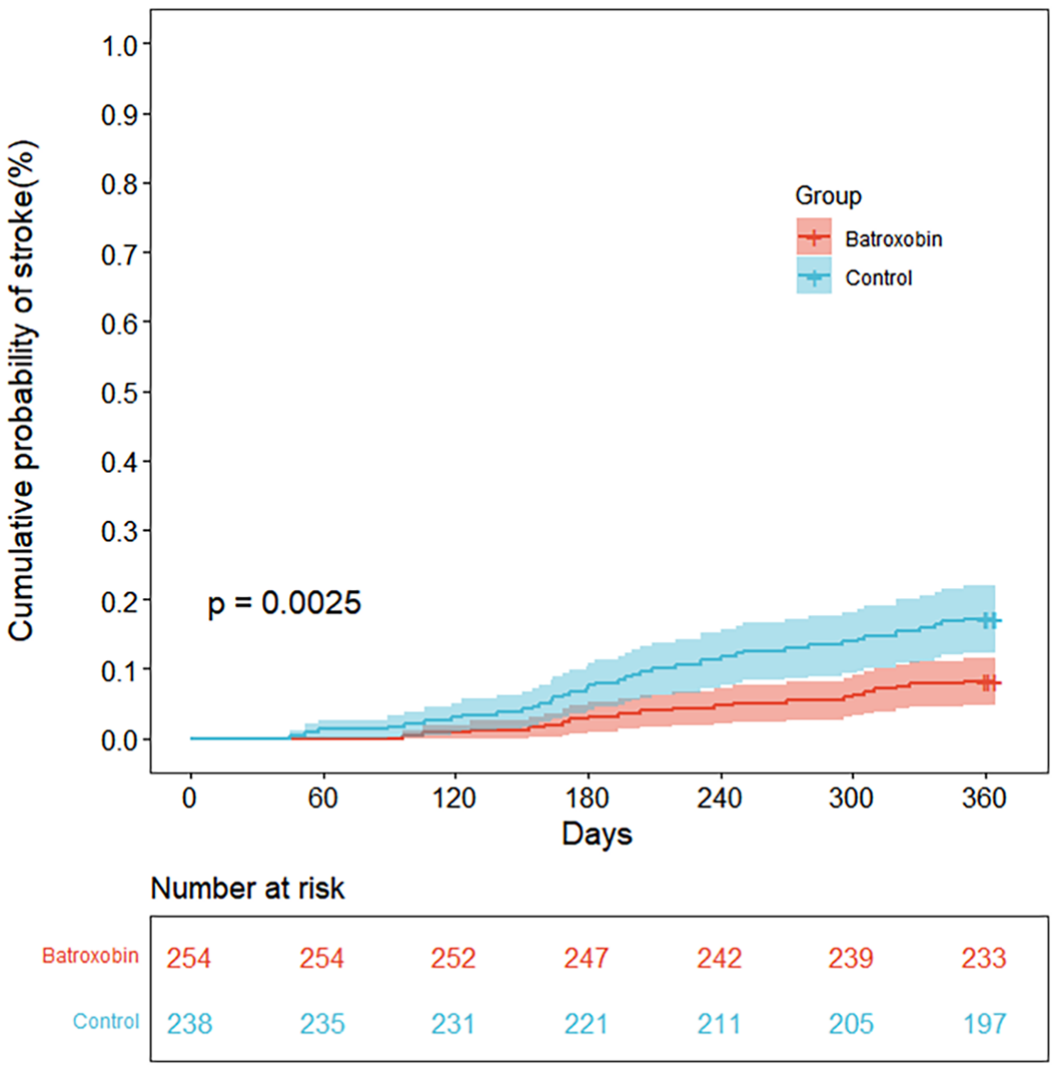
Kaplan–Meier plot for risk of stroke recurrence within 1 year.

The recurrence of ischemic stroke occurred in 21 of the 254 patients (8.3%) in the batroxobin group and 41 of the 238 patients (17.2%) in the control group within 1 year (hazard ratio, 0.433; 95% CI, 0.248 to 0.757; *p* = 0.0025) (Figure 2 and Table 2).

**TABLE 2:** Efficacy and safety outcomes.

Outcome	Group, No.(%)		HR (95% CI) or aOR (95% CI)
	Batroxobin (*n* = 254)	Control (*n* = 238)	
Primary efficacy outcome			
Recurrent stroke within 1 year	21 (8.3%)	41 (17.2)	0.433 (0.248–0.757)
Secondary efficacy outcome			
*Modified Rankin Scale score at 90‐120 days*			
0–1	144 (56.4%)	80 (33.6%)	1.536 (1.072–2.200)
0–2	179 (70.3%)	124 (52.1%)	1.362 (1.039–1.786)
0–3	198 (77.9%)	154 (64.7%)	1.195 (1.031–1.533)
Safety outcome			
Moderate‐to‐severe bleeding events within 1 year	3 (1.2%)	1 (0.4%)	2.833 (0.293–27.422)

Abbreviations: aOR, adjusted odds ratio; CI, confidence interval; HR, hazard ratio.

**FIGURE 3:**
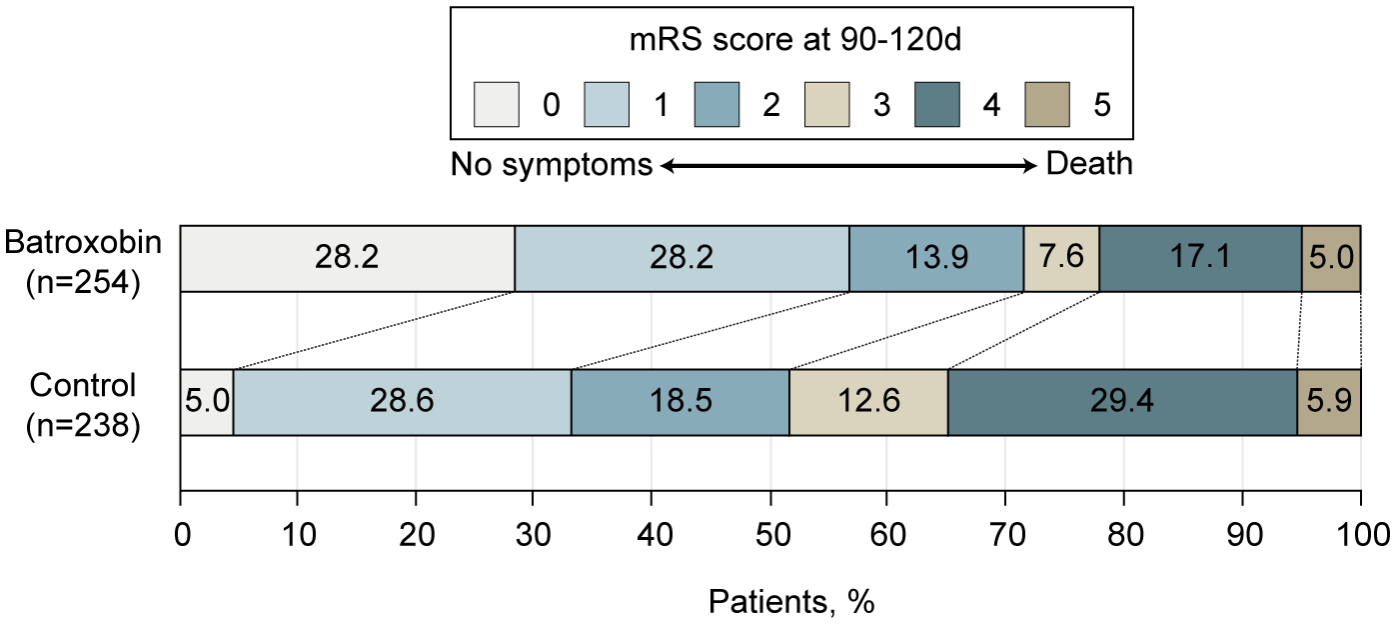
Distribution of global disability at 90–120 days based on the Modified Rankin Scale Score. Scores on the modified Rankin Scale for patients in the batroxobin group and the control group are shown. Scores on the modified Rankin Scale of functional disability range from 0 (no symptoms) to 6 (death). The score was evaluated centrally by 2 professional neurologists. MRS, modified Rankin Scale.

With respect to secondary outcomes, patients without disability (mRS score of 0–1) were 56.4% for the batroxobin group and 33.6% for the control group (difference, 22.8%; adjusted odds ratio, 1.536 [95% CI, 1.072–2.200]). Patients with functional independence (mRS score of 0 to 2) were 70.3% for the batroxobin group and 52.1% for the control group (difference, 18.2%; adjusted odds ratio, 1.362 [95% CI, 1.039–1.786]), and patients who were able to walk or take care of their own bodily needs or better (mRS score of 0 to 3) were 77.9% for the batroxobin group and 64.7% for the control group (difference, 13.2%; adjusted odds ratio, 1.195 [95% CI, 1.031–1.533]). In general, the batroxobin group demonstrated a superior functional outcomes compared to the control group (Figure 3 and Table 2).

Moderate‐to‐severe bleeding events within 1 year, as the primary safety outcome, occurred in 1 patient (0.4%) in the control group and in 3 patients (1.2%) in the batroxobin group. In both groups, moderate‐to‐severe bleeding events were not significantly different (hazard ratio, 2.833; *p* = 0.369; 95% CI, 0.293 to 27.422) (Table 2).

### 3.3 Subgroup analyses

**FIGURE 4:**
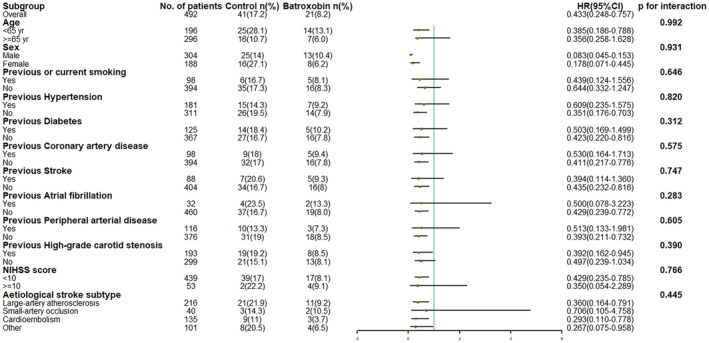
Hazard ratio for the primary outcome in prespecified subgroups. The reduction in the risk of stroke in batroxobin group, as compared with control group, was consistent across all major subgroups. There were no significant interactions in any of the 12 predefined subgroups (*p* > 0.05 for all comparisons). CI, confidence interval; HR, hazard ratio; NIHSS, National Institute of Health Stroke Scale.

The results of subgroup analyses for the primary outcome are shown in Figure 4. None of the prespecified baseline factors had significant interactions with treatment (*p* > 0.10 for all comparisons). Across all subgroups, batroxobin significantly reduced recurrent stroke (Figure 4).

## 4 DISCUSSION

In this multicenter retrospective analysis, we discovered that intravenous batroxobin therapy lowered the risk of stroke recurrence by 8.9% within a year compared to using only standard therapy in individuals dealing with acute ischemic stroke. Additionally, all prespecified exploratory subgroups showed results favoring batroxobin use, indicating that batroxobin may be a promising treatment for stroke in the future. Furthermore, intravenous batroxobin notably improved the distribution of 90–120 day disability (measured by mRS scores) among these patients. Importantly, there was not a statistically significant variance in moderate‐to‐severe bleeding events between the batroxobin and control treatment groups.

Numerous defibrinogenating agents sourced from snake venom, notably ancrod and batroxobin, have been extensively studied for their potential in treating cerebral infarction.^16^ Batroxobin primarily acts by breaking down fibrinogen into fibrin degradation products (FDPs) and D‐dimer, mobilizing endothelial cells to release native tissue type plasminogen activator (nt‐PA), thereby promoting thrombolysis. Additionally, batroxobin plays a role in exhibiting neuroprotective effects, inhibiting neuron apoptosis, reducing cerebral edema, minimizing hemorrhagic transformation, and aiding in the recovery of cerebral blood flow at the infarcted sites.^17^

Prior researches have indicated the therapeutic impact of defibrinogenating agents in acute ischemic stroke patients. In the Stroke Treatment with Ancrod Trial (STAT) of 2000, a higher percentage of patients in the ancrod group achieved favorable functional status compared to the placebo group (42.2% vs. 34.4%) within 3 h of stroke onset. Additionally, the ancrod group had a lower proportion of severely disabled patients compared to the control group (11.8% vs. 19.8%). Notably, there was no difference in mortality between both groups.^18^ Another extensive multicenter trial examining defibrase in acute cerebral infarction found significant improvements in neurological and daily living functions within 12 h of symptom onset, especially within the initial 6 h. However, defibrase administration increased the risk of extracranial bleeding events linked to plasma fibrinogen levels.^19^ Studies like the Ancrod Stroke Program (ASP) and the European Stroke Treatment with Ancrod Trial (ESTAT) suggest that the efficacy of ancrod within 6 h of symptom onset did not significantly differ from placebo in terms of functional success at 3 months.^20^, ^21^ The inefficacy observed in these trials might be attributed to treatment timing, leading to the recommendation against ancrod use beyond 3 h for acute ischemic stroke. In our investigation, batroxobin—similar to ancrod as it is derived from snake venom—showed promising results. Intravenous batroxobin infusion reduced the one‐year risk of stroke recurrence and improved neurological function at 90–120 days without elevating bleeding risk in patients with acute ischemic stroke beyond the thrombolytic time window. Our research uniquely focuses on the administration of batroxobin in a specific patient demographic—those presenting within 24 hours of symptom onset but ineligible for standard thrombolytic treatment. Similar conclusions in other studies regarding batroxobin's efficacy beyond the thrombolytic treatment window in acute cerebral infarction exist.^13^, ^14^ A broader treatment window could potentially benefit a larger cohort of acute ischemic stroke patients, presenting an opportunity for improved outcomes and optimized care.

Levated fibrinogen levels were identified as predictive of subsequent TIA or stroke.^22^ A multinational study discovered that patients with a history of TIA or ischemic stroke were at a heightened linear risk of experiencing new ischemic strokes and acute coronary events as their fibrinogen levels increased.^23^ However, a randomized trial demonstrated that there was no direct link between the degree of fibrinogen reduction and the risk of ischemic stroke. Notably, reducing fibrinogen was associated with a decreased risk of ischemic stroke in an analysis limited to patients with high baseline fibrinogen levels.^24^ In our study, the baseline fibrinogen levels were similar between the control group and the batroxobin treatment group. Therefore, the use of batroxobin in treating ischemic stroke may also be applicable to patients with normal baseline levels of fibrinogen. Additionally, we did not observe an increased risk of bleeding with batroxobin treatment, an unexpected result given the agent's defibrinogenating properties. This could be attributed to the careful selection of patients for batroxobin therapy based on stringent inclusion criteria, which may have minimized the risk of hemorrhagic complications. Moreover, this outcome prompts a reevaluation of the bleeding risks associated with defibrinogenating agents and highlights the need for personalized medicine approaches in stroke treatment. However, during the treatment process, further monitoring of fibrinogen levels is required.

The practical applications of our findings are manifold. They pave the way for the design of prospective, randomized clinical trials to confirm the benefits of batroxobin and to potentially expand its use as a standard treatment option in certain stroke populations. Furthermore, our study raises important considerations about patient selection and the timing of intervention, which are critical factors for maximizing therapeutic efficacy and safety.

Although our research uncovering these significant findings, there are a few limitations worth noting. First, the inherent limitations of the non‐randomized and retrospective design of this study necessitate further prospective clinical trials. Second, our trial was specifically conducted among Han Chinese patients, so other populations may not be directly affected by the findings. Moreover, the incidence of intracranial artery stenosis tends to be higher in Asian populations compared to non‐Asian ones.^25^, ^26^ As a result, our next step will involve investigating the effectiveness and safety of batroxobin in treating intracranial artery stenosis. Third, this trial excluded certain crucial subgroups of ischemic stroke patients, such as those with milder or more severe strokes (e.g., those with NIHSS scores <4 or >18) or those undergoing reperfusion therapy. Therefore, further research with larger sample sizes and long‐term follow‐up is needed to validate our findings.

## 5 CONCLUSION

In summary, among patients experiencing acute moderate‐to‐severe ischemic stroke within 24 h, who were outside the therapeutic window for thrombolysis, those treated with batroxobin early exhibited a reduced risk of stroke recurrence and showed improved neurological function recovery, all without an increase in bleeding events. These findings suggest a promising role for batroxobin as an alternative therapeutic approach in the management of stroke, particularly for those ineligible for standard thrombolytic therapies.

Further research is warranted to explore the long‐term effects of batroxobin treatment, optimize dosage and administration protocols, and identify subpopulations that may derive the most benefit from this intervention, and to validate these findings in larger, prospective clinical trials.

## CONFLICT OF INTEREST STATEMENT

The authors declare no competing interests.

## Data Availability

The datasets used or analyzed during the current study are available from the corresponding author on reasonable request.
